# On-demand mobile hypertension training for primary health care workers in Nigeria: a pilot study

**DOI:** 10.1186/s12913-024-10693-x

**Published:** 2024-04-09

**Authors:** Joseph Odu, Kufor Osi, Leander Nguyen, Allison Goldstein, Lawrence J. Appel, Kunihiro Matsushita, Dike Ojji, Ikechukwu A. Orji, Morenike Alex-Okoh, Deborah Odoh, Malau Mangai Toma, Chris Ononiwu Elemuwa, Suleiman Lamorde, Hasana Baraya, Mary T. Dewan, Obagha Chijioke, Andrew E. Moran, Emmanuel Agogo, Marshall P. Thomas

**Affiliations:** 1 Resolve To Save Lives, New York, USA; 2 Columbia University Irving Medical Center, New York, USA; 3 Welch Center for Prevention, Epidemiology, and Clinical Research, Johns Hopkins University, Baltimore, MD USA; 4 Department of Internal Medicine, Faculty of Clinical Sciences, University of Abuja, Abuja, Nigeria; 5 Cardiovascular Research Unit, University of Abuja Teaching Hospital, Gwagwalada, Abuja, Nigeria; 6 Federal Ministry of Health, Abuja, Nigeria; 7 National Primary Health Care Development Agency, Abuja, Nigeria; 8 World Health Organization, Abuja, Nigeria

**Keywords:** Hypertension, Online learning, Primary health care, Clinical guidelines, Mobile technology, mHealth

## Abstract

**Background:**

Only one out of every ten Nigerian adults with hypertension has their blood pressure controlled. Health worker training is essential to improve hypertension diagnosis and treatment. In-person training has limitations that mobile, on-demand training might address. This pilot study evaluated a self-paced, case-based, mobile-optimized online training to diagnose and manage hypertension for Nigerian health workers.

**Methods:**

Twelve hypertension training modules were developed, based on World Health Organization and Nigerian guidelines. After review by local academic and government partners, the course was piloted by Nigerian health workers at government-owned primary health centers. Primary care physician, nurse, and community health worker participants completed the course on their own smartphones. Before and after the course, hypertension knowledge was evaluated with multiple-choice questions. Learners provided feedback by responding to questions on a Likert scale.

**Results:**

Out of 748 users who sampled the course, 574 enrolled, of whom 431 (75%) completed the course. The average pre-test score of completers was 65.4%, which increased to 78.2% on the post-test (*P <* 0.001, paired *t-test*). Health workers who were not part of existing hypertension control programs had lower pre-test scores and larger score gains. Most participants (96.1%) agreed that the training was applicable to their work, and nearly all (99.8%) agreed that they enjoyed the training.

**Conclusions:**

An on-demand mobile digital hypertension training increases knowledge of hypertension management among Nigerian health workers. If offered at scale, such courses can be a tool to build health workforce capacity through initial and refresher training on current clinical guidelines in hypertension and other chronic diseases in Nigeria as well as other countries.

**Supplementary Information:**

The online version contains supplementary material available at 10.1186/s12913-024-10693-x.

## Background

### Hypertension in Nigeria

In Nigeria, cardiovascular disease accounts for 9% of all deaths each year [1]. Hypertension is a major driver of cardiovascular disease burden, with a prevalence between 32.5% and 38.1% among adults in Nigeria [2, 3]. However, access to care and treatment for hypertension in Nigeria is inadequate, with only 12.0–33.6% of hypertensive individuals estimated to receive treatment [2, 3]. Moreover, blood pressure control rates are extremely low, ranging from 2.8 to 12.4% [2, 3]. Several factors contribute to this situation, including poor detection and management of hypertension at primary health centers (PHCs), a shortage of health workers (HWs), limited knowledge among some HWs, and inadequate equipment and supplies at health facilities [4].

The Nigerian Federal Ministry of Health (FMOH) has established national targets and developed a roadmap to address the rising burden of non-communicable diseases based on the World Health Organization (WHO) HEARTS hypertension control technical package [5–7]. In collaboration with the National Primary Healthcare Development Agency (NPHCDA), WHO, and Resolve to Save Lives, the FMOH launched the Nigerian Hypertension Control Initiative in 2020 [8]. The initiative aims to improve population-level blood pressure control by strengthening and scaling up screening, diagnosis, treatment, monitoring, and health education at the primary health care level.

Nigeria Hypertension Control Initiative strategies include standardizing hypertension treatment with a simple treatment protocol and implementing task shifting and task sharing of hypertension service delivery duties. This approach engages all cadres of PHC staff in hypertension management. The government is currently conducting in-person training of HWs in PHCs across the country using Nigeria-specific guidelines. However, traditional in-person training has several limitations.

### Current gaps in health worker training

Low- and middle-income countries face significant challenges in establishing and maintaining a skilled health workforce to combat the growing burden of non-communicable diseases and other health challenges [9]. Conventional HW in-service training methods have not kept up with the rapid evolution of clinical guidelines and best practices [10, 11]. Nigeria has over 30,000 PHCs that are staffed by hundreds of thousands of HWs [12]. Providing in-person training to upskill the entire PHC workforce on new hypertension guidelines would be a costly and logistically challenging endeavor. The system of pre-service HW training is also under strain. Meanwhile, developing countries continue to face severe shortages of HWs, particularly in rural and underserved areas [13].

The COVID-19 pandemic has further highlighted the need for innovative approaches to HW training. The pandemic significantly disrupted traditional training methods like in-person seminars and workshops [14, 15]. Online training is emerging as a viable option to provide health professionals with the flexibility to study at their own pace and from any location while minimizing the risk of the spread of infection [14].

### Present study

Our team previously piloted and evaluated a short, mobile-optimized online infection prevention and control course with HWs in Nigeria [15]. We found that the course had high completion rates and strong learning gains. Based on the success of the online infection prevention and control course, we applied a similar methodology to train HWs based in PHCs in Nigeria on new national hypertension diagnosis and management guidelines.

## Methods

### Program design informed by learning science

We expanded on the learning approach we developed in previous pilots of an infection prevention and control course in Nigeria [15]. We used insights from the learning sciences and our understanding of HWs’ learning and technology needs to develop a set of design principles. These include:

- Structuring the learning around clinical cases that are directly relevant to HWs’ practice. This approach can boost HWs’ interest and motivation [16]. It also allows HWs to directly apply experiences and knowledge stored in long-term memory.
- Engaging HWs through continuous low-stakes assessments (quiz questions) with constructive feedback. These assessments are intended to promote learning rather than merely evaluate learners [17]. Each question is accompanied by a brief explanation, which improves learners’ subjective experience [18].
- Developing modules that repeat and expand upon key concepts, harnessing the benefits of spaced repetition to facilitate learning [19].
- Focusing on essential content and eliminating nonessential material, which improves factual retention [20].
- Teaching basic knowledge and skills, which may be more appropriate for online HW training than teaching advanced clinical practices [16].
- Offering short courses, which increases course completion [21].
- Implementing a user-friendly and well-organized learning experience, which reduces frustration and maintains learners’ self-efficacy [22].
- Requiring learners to complete a short “sample” module to enroll in the full course. Some learners who sign up for free online courses do not intend to complete them [23], so this small commitment helps to ensure that those who enroll are invested in the learning.

### Evaluation methodology

To assess short-term knowledge gains we used a pre-/post-test design. The 10-question multiple choice test was given once at the beginning of the course, with the same set of questions given again at the end of the course. Questions were presented in the same order each time, with the order of answers randomized. Although the pre-/post-test emphasized the content taught in the course, the pre-/post-test questions were not repeated in other modules of the course. Learners could only take each of these tests once and no minimum score was required on the test to advance in the course. Learners received minimal feedback (they could see the correct answers but there were no explanations given) after submitting their answers. To evaluate learners’ reactions, HWs answered a short survey at the beginning of the last module of the course. This survey included the net promoter score question, “How likely is it that you would recommend this course to a friend or colleague?” The survey also included two 5-point Likert scale questions assessing learners’ enjoyment of the course and its relevance to their work. Learners provided basic demographic data by answering a short survey at the end of the first (sample) module of the course. We based the survey questions (supplementary file 1) on questions we used in previous courses [15], with some additions and refinements to match the context of this course.

### Collaborative course development

The development of the course was a collaborative and coordinated process that involved multiple government stakeholders, academic partners, and non-governmental organizations. These entities included the FMOH, NPHCDA, WHO-Nigeria Office, Johns Hopkins University, the University of Abuja Teaching Hospital’s Hypertension Treatment in Nigeria Project team, and Resolve to Save Lives.

The FMOH coordinated the co-creation of course materials aligned with the National Hypertension Treatment Guideline, developed in 2021. Prior to the pilot study, four hypertension program managers from WHO, NPHCDA, the Hypertension Treatment in Nigeria Project, and RTSL, three FMOH policymakers, and four clinical experts reviewed the course to ensure alignment with local guidelines and cultural context. An example module from the course is provided in supplementary file 2. After the content was reviewed, the course was built on our learning management system and quality assurance was conducted by the team at Resolve to Save Lives.

Next, we conducted user testing at a PHC in Abuja to ensure the course’s usability and effectiveness. Four HWs from different cadres took part in the testing, including a medical doctor, a nurse/midwife, a community health extension worker (CHEW), and a pharmacy technician. HWs were selected who had a smartphone, an email account, and access to cellular data or Wi-Fi internet at the testing site. We carried out individual user testing sessions with each HW. Throughout these sessions, we offered an overview and context for the online hypertension course, secured consent from the participants, and clarified the procedure for accessing the course. The HW then received a text message with a link to the course and accessed selected modules on their mobile device. They were encouraged to provide feedback on their progress, including difficulties encountered, observations, and suggestions. The results of user acceptance testing were used to improve the course content and navigation for the subsequent pilot.

### Course dissemination

Enrollment in the pilot online training was open from February 13 to April 20, 2023. Learners who enrolled had access through May 4, 2023 to ensure that they had enough time to complete the course. The FMOH, NPHCDA, the Hypertension Treatment in Nigeria Project manager, and state non-communicable disease coordinators distributed the link to the online course to HWs at Nigeria Hypertension Control Initiative facilities, hypertension treatment in Nigeria project facilities, and other facilities implementing hypertension control programs. The link was primarily shared via email and WhatsApp. The target audiences included doctors, nurses, and community health workers at PHCs who care for hypertensive patients. Due to task shifting and task sharing, all of these HW cadres contribute to hypertension diagnosis and management in PHCs in Nigeria.

### Technologies used

The course was hosted on the LearnWorlds platform [24]. We optimized LearnWorlds settings to remove any unnecessary buttons or menus, simplify navigation, and maximize readability on mobile devices. Learners were required to answer all questions and move through the course in sequence. LearnWorlds was integrated with another tool, Zapier [25], to automatically enroll learners in the full hypertension course after they completed a sample module. Support requests were handled over email and learners used WhatsApp to support each other informally and contact program staff for support.

### Data analyses

All data were downloaded from the LearnWorlds platform in.csv format. To calculate pre-/post-test scores, we gave each question equal weight. The net promoter score question was presented on an 11-point scale from 0 (not at all likely) to 10 (extremely likely). We calculated net promoter score by subtracting the percentage of detractors (6 and below) from the percentage of promoters (9 and 10) [26]. Likert scale questions were numerically coded to compute a mean. All data were analyzed in Microsoft Excel. Paired *t* tests were used to evaluate learning gains.

## Results

### Enrollment, completion, and learner demographics

748 users entered the first “sample” module of the course. Of these, 574 completed the sample module to enroll in the full course. 75% of enrolled learners (*n* = 431) completed the course. The mean pre-test score was 65.4% among learners who completed the course, and 59.6% among learners who did not. Over 99% of enrolled learners reported living in Nigeria, and 75% of them were asked to take the course by a supervisor. 59% of learners reported working in a PHC that was part of an existing hypertension control program in Kano State, Ogun State, or the Federal Capital Territory. The vast majority of learners (89%) reported accessing the course on a smartphone or other mobile device. Almost all learners (98%) reported some work responsibilities related to hypertension diagnosis and management or the administration of hypertension programs (Table 1).

**Table 1:** Learners’ self-reported hypertension management responsibilities

Hypertension management task	% of learners (*n* = 574)
Measure blood pressure	88%
Prescribe antihypertensive medications	66%
Educate patients about lifestyle changes for hypertension	90%
Supervise health workers who provide hypertension care	57%
Learners who reported at least one of the above responsibilities	98%

70% of learners reported completing training for at least one clinical role (Junior CHEW, CHEW, community health officer, Nurse, Midwife, or Doctor). 54% of learners reported completing some post-secondary schooling (but not a bachelor’s degree), and 34% reported completing a bachelor’s degree.

### Learning gains and completion by health worker cadre and education level

**Fig. 1:**
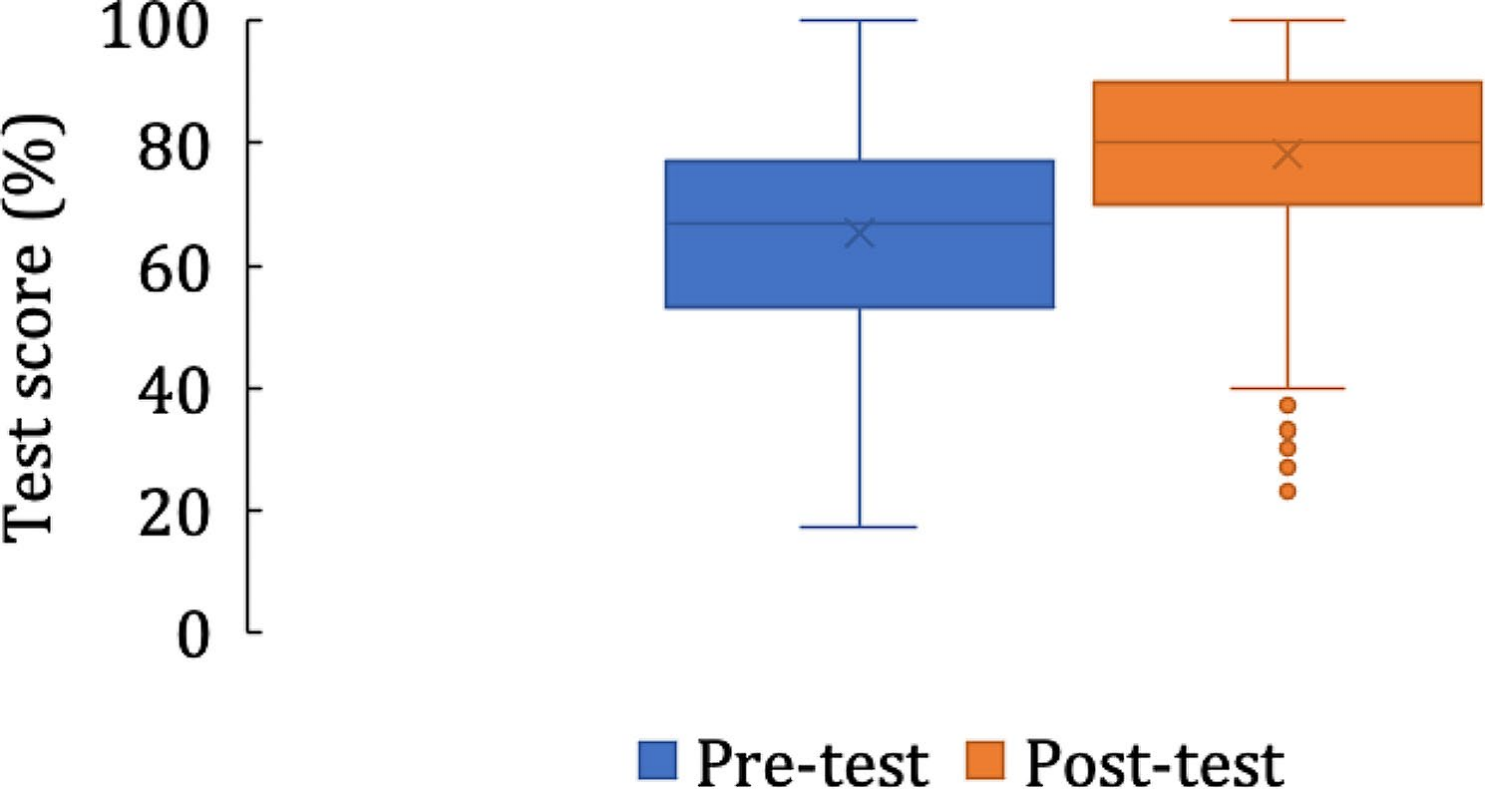
Pre- and post-test scores among learners who completed the course (*n* = 431)

Among the 431 learners who completed the course, the pre-test score was 65.4% and the post-test score was 78.2% (*P <* 0.001). There was a wide range of scores on both tests (Fig. 1).

There were differences in test scores and course completion by HW cadre (Table 2), but learning gains were significant in all cadres. Nurses, midwives, doctors, and those who completed a bachelor’s degree or above in microbiology or biomedical sciences had the largest learning gains. Doctors had the highest pre-test scores of any group, while community health officers had the lowest pre-test scores. CHEWs had the highest completion percentage and doctors had the lowest completion percentage.

**Table 2:** Course completion and learning gains by HW cadre

	Enrolled participants (*n* = 574)		Completed learners (*n* = 431)		
Training for roles	% of total	% completion	Pre-test	Post-test	Δ
CHEW or junior CHEW	42%	79%	64.1%	73.8%	+ 9.7%
Community health officer	11%	76%	58.7%	71.6%	+ 12.9%
Nurse	17%	72%	63.2%	81.7%	+ 18.5%
Midwife	13%	75%	63.7%	80.0%	+ 16.3%
Doctor or medical officer	7%	54%	69.8%	86.9%	+ 17.1%
Degree in microbiology / biomedical sciences	15%	76%	65.8%	84.8%	+ 19.0%
None of the above	21%	74%	68.3%	80.6%	+ 12.3%

Learners could select ≥ 1 roles. Differences in pre- and post-test scores were statistically significant within each HW cadre (*P* < 0.001; paired *t* test).

### Learning gains interact with study time and prior participation in hypertension control programs

Learners who completed all learning activities spent a median time of 160 min working in the course. Those who took longer to complete the course had greater learning gains than those who spent the median amount of time or less (Table 3). 59% of the 431 learners who completed the course reported working in a PHC that was part of an existing hypertension control program. These learners had higher pre-test scores and lower learning gains than those who were not working at such PHCs (Table 3).

**Table 3:** Learning gains by time spent in the course and work status at PHCs participating in an existing hypertension control program (*n* = 431)

Learner subgroup	Pre-test	Post-test	Δ
Median or below time spent in the course	65.7%	74.9%	+ 9.3%
Above median time spent in the course	65.1%	81.5%	+ 16.4%
Staff at PHC in an existing hypertension program	68.3%	77.1%	+ 8.9%
Not staff at a PHC in an existing hypertension program	60.6%	79.9%	+ 19.3%

Learning gains were significant (*P* < 0.001) within each group.

### Learner feedback

Of 434 responses to the net promoter score question, the average rating was 9.6/10, corresponding to a net promoter score of + 86. Learners also gave the course high ratings on two Likert-scale questions assessing their enjoyment of the course and its relevance to their work (Table 4).

**Table 4:** Learner feedback on the course

	I enjoyed this training (mean: 4.83)		This training was relevant to my work (mean: 4.76)	
	N	%	N	%
Strongly agree	361	83.2%	358	82.3%
Agree	72	16.6%	60	13.8%
Neutral	0	0%	6	1.4%
Disagree	1	0.2%	11	2.5%
Strongly disagree	0	0%	0	0%

The number of respondents and percentage of total is reported for each response. Responses were numerically coded (strongly disagree– 1; strongly agree– 5) to calculate an average score.

## Discussion

We developed and piloted an online learning approach to train HWs in Nigeria on new national guidelines for hypertension management. The pilot had a high percentage of completion, positive learner feedback, and significant learning gains across different categories of PHC-based HWs. Most importantly, learners significantly gained clinically relevant knowledge regardless of their cadres. Along with our previous work [15], these results suggest that a mobile, digital, on-demand training approach is effective for training PHC-based HWs in Nigeria on best clinical practices in hypertension management. Future research, ideally randomized controlled trials, will be needed to determine the impact of such training on patient care and health outcomes.

This course had a high percentage of completion compared to industry norms, with 75% of enrolled learners completing the course, in contrast to massive open online courses, in which completion rarely exceeds 25% [27–30]. The high percentage of completion of this course is likely due to its endorsement by the FMOH and the encouragement HWs received to take the course from their supervisors. Factors that affect online course completion include endorsement and promotion by employers, the government, trusted sources, and professional networks [31–33]. Other factors reported to promote uptake of online courses include perceived usefulness and value relative to the effort required [29, 34, 35]. We hypothesize that the case-based nature of the training, its brevity, and its focus on only the most relevant material contributed to its effectiveness, as more than 95% of HWs said the course was relevant to their work, and almost all reported performing at least one work task related to hypertension management.

Learning gains were substantial and statistically significant, indicating that HWs’ knowledge of hypertension management improved upon completing the course in all relevant cadres. However, we observed some heterogeneity of learning gains, with HWs who had completed four or more years of post-secondary education (such as nurses, midwives, and medical doctors) having greater learning gains than cadres of HWs with two or three years of post-secondary schooling (CHEWs and community health officers). This association between level of formal schooling and preparedness to succeed in self-paced and self-directed online learning is in keeping with several previous studies, which reported the importance of self-efficacy, motivation, and digital skills to a participant’s success in online learning [33, 36–39].

HWs who were not staff at PHCs taking part in existing hypertension treatment programs had lower pre-test scores and greater learning gains than those who were. This is at variance with other studies, which suggested that low prior knowledge is associated with poor outcomes in online courses [40–42]. It may be that those who were not in existing hypertension programs were more motivated to learn hypertension management, thereby leading to the large knowledge gains we observed in this group. Therefore, this mobile training approach might be useful for introducing HWs to new guidelines while also acting as “refresher training” for HWs who have already learned new guidelines.

Learner feedback was overall positive. Responses to two Likert-scale questions indicated that most learners found the course enjoyable and relevant to their work, with higher ratings than similar questions reported in other courses [43–45]. The net promoter score of this course (+ 86) was high, exceeding the “excellent” benchmark of + 50 and the reported net promoter score of other online courses [15, 46, 47].

On-demand, smartphone-based health worker training has the potential to fill the demand for clinical training efficiently and at lower cost compared with traditional, in-person training in Nigeria and other countries. The Nigeria hypertension training course can be deployed to meet several hypertension control program needs: (1) induction training of workers either *en masse* at facility activation, or when new workers join primary care teams that were previously trained, or (2) refresher training of workers who were already trained.

Online training for HWs is becoming more widespread globally due to higher demand (more HWs who can access digital training) and increasing supply (more options for those learners). The rapid rise in access to cellular internet and increasing smartphone ownership in Africa [48] could be a fulcrum that will promote online training in this part of the world. In Nigeria, an estimated 55% of the population is connected to the internet and over 97% of users access the internet with a mobile device [49]. Most HW cadres that work in PHCs in Nigeria have some post-secondary education, and many have completed bachelor’s degrees, so this group may be among early adopters of technology-enabled learning. It remains unclear whether mobile training applications are best used as an adjunct to traditional in-person group training, or a substitute for in-person training. Finally, rigorous educational and economic evaluations are needed to inform the optimal strategy for primary care health worker training.

### Limitations

We only evaluated short-term knowledge gains, and we haven’t yet tested the impact of this training on clinical skills (such as proper blood pressure measurement) or clinical outcomes (such as control of hypertension). Without a comparator group, we can’t be certain that the observed knowledge gains are fully attributable to the online course. All data on learners’ demographics and education levels are self-reported and we did not independently verify the identity of course participants. Many HWs were asked to take the course by their supervisors and were encouraged to complete the course within 14 days, which may have led some HWs to rush through the course to complete it. There was also likely self-selection of HWs with high motivation, access to an internet-connected smartphone, and a certain level of digital literacy and familiarity with navigating through interactive websites.

## Conclusions

We found that a simple-to-use, mobile-optimized, case-based online short course can effectively train PHC-based HWs on updated hypertension management guidelines. A high percentage of learners completed the course, learner feedback was very positive, and there were significant learning gains in all cadres of HWs. These results suggest that such training is a scalable way to build health workforce capacity on new clinical guidelines and to refresh knowledge of best clinical practices. Either on its own or in combination with traditional in-person group trainings, this approach could be applied to a variety of topics to improve HWs’ adherence to evidence-based practices in Nigeria and elsewhere.

### Electronic supplementary material

Below is the link to the electronic supplementary material.

Supplementary Material 1

Supplementary Material 2

## Data Availability

De-identified data are available from the corresponding author upon request.

## Abbreviations

CHEW: community health extension worker
FMOH: Federal Ministry of Health
HW: health worker
NPHCDA: National Primary Health Care Development Agency
PHC: primary health center
