# Supplementary material for: On-demand mobile hypertension training for primary health care workers in Nigeria: a pilot study
Source: BMC Health Serv Res. 2024 Apr 9;24:444. doi: 10.1186/s12913-024-10693-x (PMC11005121; doi:10.1186/s12913-024-10693-x)
Supplement: Supplementary file 1 — Supplementary Material 1 [file 12913_2024_10693_MOESM1_ESM.pdf]

Pre-course survey

Do you live in Nigeria?
Yes
No

Do you currently work in a primary health facility in Kano or Ogun State that is part of the Nigeria Hypertension Control Initiative?
Yes
No

Do you currently work in a primary health facility in Abuja that is part of the Hypertension Treatment in Nigeria Program?
Yes
No

What is the highest level of school/education you have completed?
Primary
Secondary
Post-secondary certificate course
Ordinary national diploma
Higher national diploma
Bachelor's degree
Master's degree
Doctoral degree (Ph.D.)

Have you completed training for any of the following positions? (select all that apply)
Junior Community Health Extension Worker
Community Health Extension Worker
Community Health Officer
Nurse
Midwife
Doctor or Medical Officer
Degree (bachelor's or above) in microbiology or biomedical sciences
None of the above

Do you perform any of the following tasks? (select all that apply)
Measure blood pressure
Prescribe antihypertensive medications
Educate patients about lifestyle changes for hypertension
Supervise health workers who provide hypertension care
None of the above

What type of device are you using to access this training?
Smartphone or other mobile device
Laptop or desktop computer
Tablet computer

Did your supervisor or the leadership of your health facility ask you to take this course?
Yes
No

End-of-course survey

How likely is it that you would recommend this training to a friend or colleague?
10 – Extremely likely
9
8
7
6
5
4
3
2
1
0 – Not at all likely

I enjoyed this training.
Strongly agree
Agree
Neutral
Disagree
Strongly disagree

This training was relevant to my work.
Strongly agree
Agree
Neutral
Disagree
Strongly disagree

What was the **best** part of this training?
[Open response]

What is **one way** in which this training could be improved?
[Open response]
