# Supplementary material for: On-demand mobile hypertension training for primary health care workers in Nigeria: a pilot study
Source: BMC Health Serv Res. 2024 Apr 9;24:444. doi: 10.1186/s12913-024-10693-x (PMC11005121; doi:10.1186/s12913-024-10693-x)
Supplement: Supplementary file 2 — Supplementary Material 2 [file 12913_2024_10693_MOESM2_ESM.pdf]

How to read this document:
This is one module from the Hypertension in Nigeria online course. The module presents a clinical case. Learners move through the module in sequence. Each new "page" in the module is indicated with the "~" symbol. Pages can contain text, images, video, or questions about the case.

Many of the questions are multiple choice or "checkboxes" questions. For these questions, the correct answers are indicated in **bold**.

After answering each question, learners saw feedback indicating the correct and incorrect answers, along with an explanation.

Instructions that aid reading of this document, but were not shown to users, are enclosed by brackets, e.g., [Feedback].

The module text begins below.

~Module 7: Ibrahim's follow-up visit
Welcome to Module 7: Ibrahim's follow-up visit. This module should take about 10-20 minutes to complete.

Ada is a nurse at a primary health center (PHC) in Kano state. Her health center recently conducted a community screening program for high blood pressure (BP). Ada supervises Femi, a community health extension worker.

Ibrahim is a 37-year-old man. His BP was taken as part of the community screening program. At that time, he had a BP reading of 144/82 mmHg. He was asked to come to the PHC in one week for follow-up.

Abbreviations used in this module:
BP - blood pressure
PHC - primary health center

~A first measurement at a follow-up visit
[This is a "matching" question type – users had to drag and drop the numbers to match – the correct answer is shown below]
Ibrahim arrives at the PHC, where Femi takes his BP. Ada confirms that the measurement is taken correctly.

The first reading in this follow-up visit is **148/94** mmHg.

Use the menu below to match the type of pressure to the reading.

Diastolic blood pressure | 94
Systolic blood pressure | 148

[Feedback]
The higher number is **systolic** BP and the lower number is **diastolic** BP.

~Factors that Influence BP
Which of the following could cause an **incorrect BP measurement**? (select all that apply)

The patient smoked a cigarette in the last 30 minutes.
The patient drank coffee in the last 30 minutes.
The patient drank water in the last 30 minutes.
The patient exercised in the last 30 minutes.
The patient's BP was taken immediately upon arrival at the clinic without a chance to rest.

[Feedback]
The patient should not **drink coffee or tea**, **exercise**, or **consume tobacco** in the 30 minutes before the BP reading. These activities could lead to a high reading that is not correct. In addition, the patient should rest for at least five minutes after arriving at the clinic and must also be positioned properly.

~Proper BP measurement
Please review the figure below that shows the proper way to measure a patient's BP.

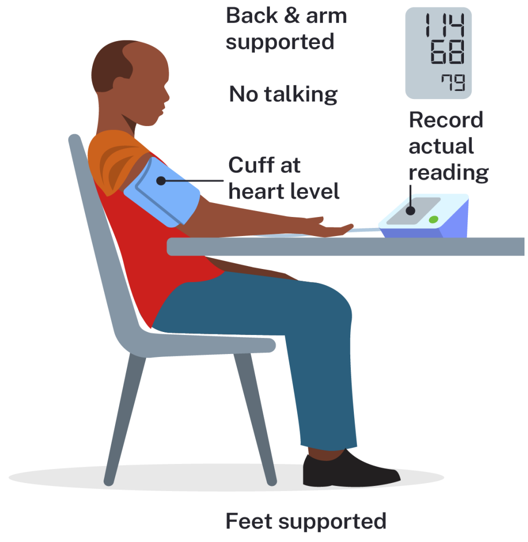

~Femi's next steps

Femi has now taken Ibrahim's BP once, and the reading was 148/94 mmHg. Femi is not sure what to do next. He asks Ada for advice and she says he should:

Repeat the BP measurement
Refer Ibrahim to a doctor for follow-up
Provide Ibrahim with lifestyle counseling
Start Ibrahim on 5 mg amlodipine per day
Start Ibrahim on 5 mg amlodipine + 50 mg losartan per day

[Feedback]
This is the first time Ibrahim has had his BP measured in this encounter and his BP is ≥140/90 mmHg.

Femi should wait three to five minutes and repeat the BP measurement.

~A second measurement on the same day

Femi waits for three minutes and repeats the BP measurement. The new BP reading is **145/90** mmHg. He is worried because the measurement is different from the first reading of **148/94** mmHg. He asks Ada if he should take a third reading.

What should Femi do next?

He should repeat the readings until he has two that match.
He should use the first reading.
He should use the second reading.

[Feedback]
The readings are quite close, and a second reading is often lower than the first. Femi can confirm a diagnosis of hypertension and use the second reading.

~Diagnosing hypertension
This short video covers some of the key concepts you have learned so far about diagnosing hypertension. (Source: Global Hypertension at Johns Hopkins)

[This video was embedded in the module: https://youtu.be/c8gL5ZGKRxc]

~Femi's recommendations

At this point, we know that:

- Ibrahim's BP was measured one week ago. At that time, his BP was **144/82** mmHg.

- Femi has confirmed that Ibrahim has a BP of **145/90** mmHg at this visit.

According to the Nigeria Hypertension Treatment Protocol, what should Femi do next? (**select two answers**)

Repeat the BP measurement
Refer Ibrahim to a doctor for follow-up
Provide Ibrahim with lifestyle counseling
Start Ibrahim on 5 mg amlodipine per day
Start Ibrahim on 5 mg amlodipine + 50 mg losartan per day

[Feedback]
Ibrahim has high BP (≥140/90 mmHg) which has been confirmed on two different days. He can start treatment for hypertension.

He should begin with 5 mg of amlodipine per day, according to the Nigeria Hypertension Treatment Protocol

~The Nigeria Hypertension Treatment Protocol
The Nigeria Hypertension Treatment Protocol is shown below.

Measure blood pressure of **all adults** ≥ 18 years of age.

High blood pressure: SBP ≥ 140 mmHg or DBP ≥ 90 mmHg.

Step 1
If BP ≥140/90 mmHg,*
Start amlodipine 5 mg.

Step 2
After 1 month, measure BP again. If still high,
Treat with amlodipine 5 mg + losartan 50 mg.

Step 3
After 1 month, measure BP again. If still high,
Treat with amlodipine 10 mg + losartan 100 mg.

Step 4
After 1 month, measure BP again. If still high,
Treat with amlodipine 10 mg + losartan 100 mg + HCTZ 25 mg.

Step 5
After 1 month, measure BP again. If still high,
Refer for specialist hypertension management.

~Ibrahim's treatment

Femi prescribes Ibrahim **5 mg amlodipine per day**. What class of medication is amlodipine?

Angiotensin converting enzyme inhibitor (ACE-I)
Angiotensin receptor blocker (ARB)
Beta blocker
Calcium channel blocker
Thiazide diuretic

[Feedback]
Amlodipine is a **calcium channel blocker**.

~Helping Ibrahim

What are some things that Femi should do next to **help Ibrahim remember to take his medication**?

[open response – user responses to these questions are not scored – after they answer, they are brought to the feedback page that follows]

~Helping Ibrahim
There are many things that Femi can do to help Ibrahim take his medication:

- Make sure that Ibrahim has at least a one-month supply of amlodipine,
- Set an appointment for Ibrahim to come back in one month,
- Text Ibrahim to check in on him and remind him of his follow-up appointment,
- Make sure that Ibrahim has a routine for taking his medication, promoting its integration into regular activities. For example, he might take the medication when he first wakes up every morning, or at the time of prayers, meals, or TV/radio shows. He can also set up alarms/reminders on cell phones, watches/clocks, or calendars, and
- Teach Ibrahim about the asymptomatic nature and long-term health impacts of hypertension.
- Explain that hypertension can be controlled, not cured, and requires lifelong therapy with medications and lifestyle changes.

~Educating Ibrahim

Which of the following are complications that a patient can experience due to uncontrolled high BP? (select all that apply)

Heart attack
Stroke

Kidney failure
Blindness

[Feedback]

Patients could experience any of these issues due to high BP.

~Summary: Ibrahim's follow-up visit

Here are some key things that you learned:

- There is a lot of natural variation in BP.
    - If the first reading in an encounter is **≥140/90** mmHg, take a second reading.
    - If the two readings are close (within 5 mmHg), use the second reading.
    - If the two readings are not close, take a third reading and use that reading.
- Hypertension is defined as sustained and elevated:
    - systolic BP **≥140** mmHg, or
    - diastolic BP **≥90** mmHg.
- Most cases of hypertension can be treated at PHCs using the Nigerian Hypertension Treatment Protocol.
- Most patients with a diagnosis of hypertension should begin treatment right away once the diagnosis is confirmed. Some patients, such as those who are pregnant, should be referred to a higher level of care for hypertension management.

Common classes of medications used to treat hypertension

**Medication class**	**Example used in Nigeria Hypertension Treatment Protocol**
Calcium channel blocker	Amlodipine
Angiotensin receptor blocker (ARB)	Losartan
Thiazide-like diuretic	Hydrochlorothiazide (HCTZ)

References
Nigeria Hypertension Treatment Protocol
Nigeria Hypertension Control Initiative Manual – Chapter 2
